# The Cobweb of Life Revealed by Genome-Scale Estimates of Horizontal Gene Transfer

**DOI:** 10.1371/journal.pbio.0030316

**Published:** 2005-08-30

**Authors:** Fan Ge, Li-San Wang, Junhyong Kim

**Affiliations:** **1**Department of Biology, University of Pennsylvania, Philadelphia, Pennsylvania, United States of America; University of TexasUnited States of America

## Abstract

With the availability of increasing amounts of genomic sequences, it is becoming clear that genomes experience horizontal transfer and incorporation of genetic information. However, to what extent such horizontal gene transfer (HGT) affects the core genealogical history of organisms remains controversial. Based on initial analyses of complete genomic sequences, HGT has been suggested to be so widespread that it might be the “essence of phylogeny” and might leave the treelike form of genealogy in doubt. On the other hand, possible biased estimation of HGT extent and the findings of coherent phylogenetic patterns indicate that phylogeny of life is well represented by tree graphs. Here, we reexamine this question by assessing the extent of HGT among core orthologous genes using a novel statistical method based on statistical comparisons of tree topology. We apply the method to 40 microbial genomes in the Clusters of Orthologous Groups database over a curated set of 297 orthologous gene clusters, and we detect significant HGT events in 33 out of 297 clusters over a wide range of functional categories. Estimates of positions of HGT events suggest a low mean genome-specific rate of HGT (2.0%) among the orthologous genes, which is in general agreement with other quantitative of HGT. We propose that HGT events, even when relatively common, still leave the treelike history of phylogenies intact, much like cobwebs hanging from tree branches.

## Abstract 2

A stastical approach applied to 297 orthologous gene clusters in 40 microbial genomes suggests a low rate of interspecies gene transfer. Species relationships can therefore be modeled with a tree structure.

## Introduction

The role of horizontal gene transfer (HGT) in speciation, adaptation, and evolution of life on earth has been studied intensively [1], and there has been a growing body of evidence of transfers of genes among species [2–4] and transfers from organelles to nuclei [5–7]. Whole genome analyses of different prokaryotes have been thought to indicate rampant HGTs [8,9] and suggest that HGT plays a pivotal role in prokaryotic evolution, producing dynamic and mosaic genomes. The speculation [10] that even genes involved in transcription and translation might have been subject to HGT has also led to the suggestion that HGT should be considered the essence of phylogeny and that HGT might have eroded the organismal genealogical trace. Therefore, life history cannot be properly represented by the traditional treelike form, but rather by a netlike form [4,11–13].

One of the main unresolved issues in the debate is the estimation of HGT frequency [14] and its impact on phylogeny [15]. Commonly used methods for detecting HGT are based on observations of (1) atypical gene sequence composition [16,17]; (2) unexpected rankings for sequence similarity among homologs [18]; and (3) incongruence among phylogenetic trees [e.g., 7]. Studies based on sequence characters suggested HGT frequency at 24% in *Thermotoga* [2] and a range up to 17% among different prokaryotes [19,20]. Conflicts between the 16S rRNA tree and other gene trees have been frequently reported [e.g., 21]. These findings have led to the ongoing debate about the impact of HGT on phylogeny. Some researchers believe that HGTs are so frequent that a core of nontransferable genes might not exist and that phylogeny in treelike form has little utility [20,22]. Other researchers, however, believe that HGTs constitute only minor interference when inferring phylogeny and propose that methods for inferring HGT have various problems leading to an overestimation of its frequency [1]. For example, a previous study shows that different methods for estimating HGT gave different sets of HGT candidates when applied to the same genome [23]. Meanwhile, it has been proposed that a phylogeny could be sufficiently retrieved via a core of genes that may be resistant to HGT [24,25]. A congruent phylogenetic structure inferred from different genes was proposed as further evidence to buttress this argument [26–29].

As pointed out in Daubin and Ochman [30], there is a difference between assessing genetic transfer among elements with some recognizable homology or orthology to sequences in other genomes and assessing genetic transfer in the entire genome, which may have indeed incorporated significant foreign genetic material, through processes such as selection for pathogenicity [19]. Thus, the key question is whether sequences with recognizable homologs in a significant number of genomes show high levels of HGT and whether HGT's effects are sufficient enough to impede the building of branching phylogenetic history [31]. HGT events lead to incongruent phylogenies for different genetic elements. But at the same time, incongruence in phylogenies can be caused by a list of factors, such as artifacts of phylogenetic reconstruction or other biological sources [32,33]. The inference of HGT from tree comparisons should be done under a proper statistical framework [14]. Furthermore, though HGT events may have occurred with high frequency in genome evolution, perhaps even affecting every gene somewhere in the tree of life, if these events are randomly distributed across the lineages and do not involve more than 50% of the genome at a time, there still exists a backbone tree structure that best fits the majority of the genome. That is, a treelike history will continue to be the most predictive representation of the whole genome and reflect the major mode of genetic information transfer [34], while the HGT events will constitute minor information exchange, much like cobwebs on tree branches. This treelike history of the majority of the genome, which we will call the whole-genome phylogeny, has two utilities. First, barring truly rampant genetic admixture, the tree represents a hypothesis about the major flow of genetic information mediated by the cell replication lineages. Second, such a tree can provide a backbone estimate, from which individual HGT events can be estimated.

Here, we first extend the approach of Lerat et al. [28] and Novichkov et al. [35] with a new method to explicitly test for phylogenetic incongruence due to horizontal transfer versus statistical tree errors, and we subsequently apply it to a larger diversity of genomes. The specific questions we ask in this paper are (1) What is the fundamental structure of the whole-genome (W-G) tree? (2) How do individual gene trees differ from this tree, especially in terms of putative HGT events? (3) How do individual gene trees differ from one another in terms of HGT? (4) What is the rate of HGT events per genome and what kind of genome-specific patterns or gene-specific patterns are evident for HGT events? To answer these questions, we used the Clusters of Orthologous Groups (COG) database of the National Center for Biotechnology Information [36] and extracted the most reliable orthologous clusters. A gene tree was built for each reliable COG and was also integrated to construct a W-G tree. Then, this tree was compared with each gene tree to infer significant HGT events based on our new statistical procedure. We augmented this procedure with a pairwise comparison of gene trees to each other to detect conflicting gene trees. Overall, we find a relatively small proportion of the COG entries with significantly detectable HGT events.

## Results

Figure 1 shows our computational flow, which we briefly describe here and in detail in the Materials and Methods section. First, we use the COG database to assemble a set of high-quality orthologous groups for tree inference. Then we use both the combined information in all of the orthologous sequences and the method of Kim and Salisbury [34] to construct the W-G tree that represents the best treelike description for the genealogical relationship of the genomes. Next, we estimate a tree for each orthologous group (which we will refer to as a gene tree) and assess the difference between the tree structure of each gene tree and that of the W-G tree. Next, we augment this comparison with all pairwise comparison of the gene trees. Finally, we evaluate the differences in the tree structure to determine whether these differences can be statistically explained away as general tree errors (e.g., due to noise in the data) or as a set of putative horizontal transfer events. The key point in this procedure is that we explicitly test the alternative hypothesis of HGT rather than merely a rejection of congruence. In the end, for those genes that display statistically significant evidence of horizontal transfer, we estimate the position of the transfer events based on the W-G tree and compute genome-specific and gene-specific rates of transfer.

**Figure 1:**
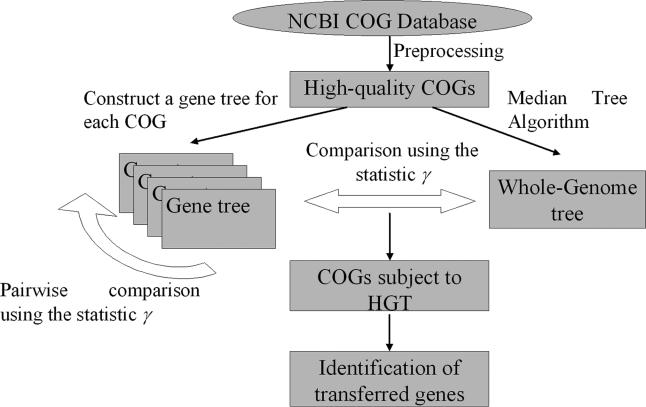
Flowchart of the HGT Inference Procedure The National Center for Biotechnology Information COG database is preprocessed for a high-quality COG set. This set is used to construct individual gene trees and the W-G tree, using the median tree algorithm. The gene trees are compared against the W-G tree to detect changes in tree topology that are best explained by a branch transfer. The same comparison is done among all gene trees.

### High-Quality Gene Groups and the W-G Tree

The COG database, built by all-versus-all sequence comparisons, covers 43 microorganisms, including complete genomes of bacteria, Archaea, and *Saccharomyces cerevisiae,* in the initial version, which we used for this study. Our stringent high-quality COG selection procedure described in the Materials and Methods section resulted in the retention of 297 COG entries out of the original 3,852, which cover 40 genomes (Table 1). On average, each high-quality COG covered 16.5 genomes, representing both universally distributed genes and lineage-specific genes. Rather than use any single set of sequences (e.g., rRNA) to approximate the W-G tree, we used the median tree estimator designed by Kim and Salisbury [34], which is a robust estimator that attempts to overcome major genetic distortions such as HGTs. The high-quality COG entries were used to construct the median tree estimate (as shown in Figure 2) with bootstrap values obtained from bootstrap resampling of the input COG entries (the branches with less than 50% bootstrap support were collapsed to improve the reliability of later analysis).

**Figure 2:**
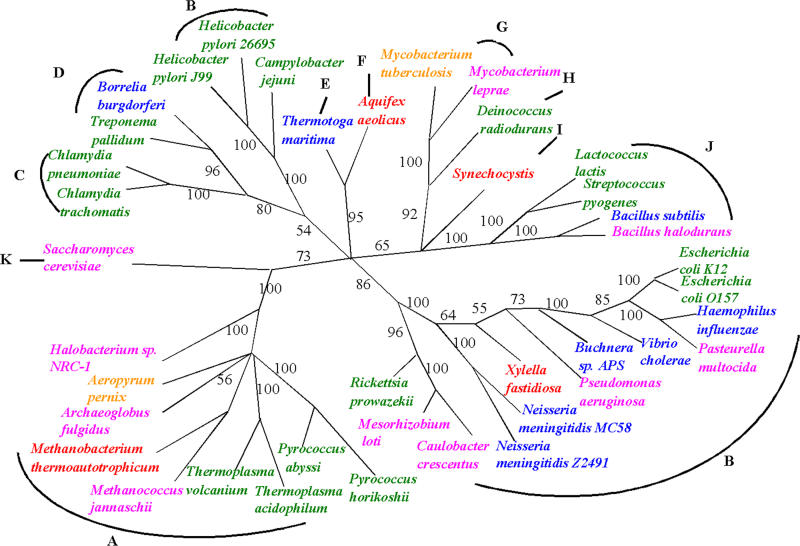
The W-G Tree Based on the Median Tree Algorithm A subset of high-quality COG entries, which covers at least seven genomes, was used to build the W-G tree (see Materials and Methods). Branches with bootstrap scores less than 50% were collapsed into the polytomous form. Three domains of life are shown as (A) Archaea, (B–J) Bacteria, and (K) Eukaryote. Species are labeled with different colors based on their inferred HGT rates: red, >4%; yellow, 3%–4%; pink, 2%–3%; blue, 1%–2%; green, <1%. Taxonomy labels are (A) Euryarchaea, (B) Proteobacteria, (C) Chlamydiae, (D) Spirochaetes, (E) Thermotogae, (F) Aquificae, (G) Actinobacteria, (H) Deinococcus, (I) Cyanobacteria, (J) Firmicutes, and (K) Fungi.

**Table 1:**
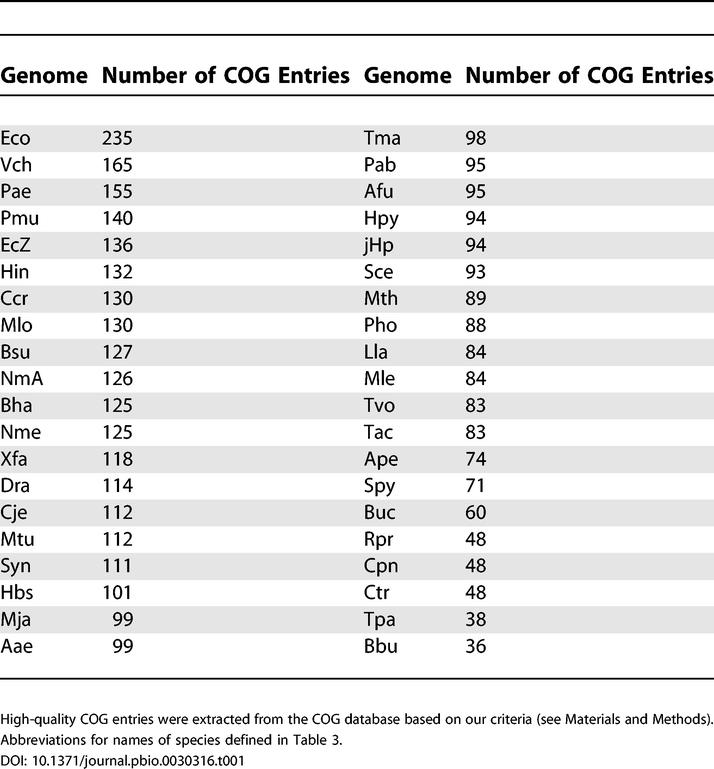
Number of COG Entries That Contain Each of 40 Genomes

In this unrooted W-G tree, the three domains of life are monophyletic with high bootstrap values. Also, the tree strongly supports the monophyly of *Chlamydiales,* Spirochaetes, low G+C gram-positives, high G+C gram-positives, and α-, β-, γ-, and δ-Proteobacteria. The artifactual attraction of long branches of Archaea and hyperthermophilic bacteria does not appear, with the grouping of *Aquifex aeolicus* and *Thermotoga maritima* into the bacterial domain, which is consistent with recent studies [37]. However it should be noted that other authors suggest *A. aeolicus* should group with Proteobacteria based on shared putative unique indels, protein domain architecture, and membrane structure [38–41], and the grouping remains controversial. Although most of the branches are supported by high bootstrap values, it is worth noting that this tree is partially unresolved, as branches with bootstrap values lower than 50% have been collapsed. Hence, this tree neither informs us on the basal position of the bacterial domain, nor informs us much on the basal branching patterns of archaeal phylogeny, which results in some loss of power for detecting HGT events across these lineages (see also Discussion). Outside of this, two possible artifacts are the basal position of *Halobacterium* at the archaeal domain, which has been suggested to be affected by a large number of HGT events from bacterial origin [42,43], and the grouping of ɛ-Proteobacteria with *Chlamydiales* and Spirochaetes, which are commonly seen in literature [27].

### Statistical Inference of HGT and Power Test

HGT events for a particular gene or sequence can be detected using phylogenetic methods that compare the estimated gene tree for the candidate sequences against the other gene trees or against some candidate tree that represents the history of the genomes. Mathematical tree distance metrics can be used to measure the discrepancy between two trees. However, two trees may be different because of an HGT event or other reasons, such as noise in the data, compositional bias, hidden gene duplication, gene loss, and so on. Thus, one possible approach is to ask whether the discrepancy between two trees can be more easily explained by simple branch-exchange events (which would be evidence of HGT)—i.e., to explicitly consider the HGT as an alternative hypothesis.

Suppose we have two trees, A and B. If A and B differ by branch-transfer events, they should share a common subtree, wherein the transferred branches have been removed. A bound on the size of such a shared common subtree can be computed using an algorithm called maximum agreement subtree (MAST [44]). Moreover, the difference between A and B can also be measured by the number of branch edges shared by the two trees, computed by a measurement called symmetric difference (SD) metric (also known as Robinson-Foulds metric [45]). Simply, the SD metric computes the number of different splits, regardless of whether or not the difference in the two trees can be explained by a branch switch (and thus putative HGT). Hence, the combination of MAST distance and SD distance between tree A and tree B can be interpreted in terms of putative HGT events (Figure 3). If both MAST and SD distance values are low, then the two trees are not likely to be statistically different. If both MAST and SD values are large, then they may be different, but the difference is not easily explained by an HGT event. The disparity could be due to many other factors (including, of course, HGT). On the other hand, if the two trees differ by a large SD value but are generally similar with a small MAST score, this suggests that the difference can be best explained by putative HGT events. The last case, large MAST distance but low SD distance, cannot occur due to algorithmic reasons.

**Figure 3:**
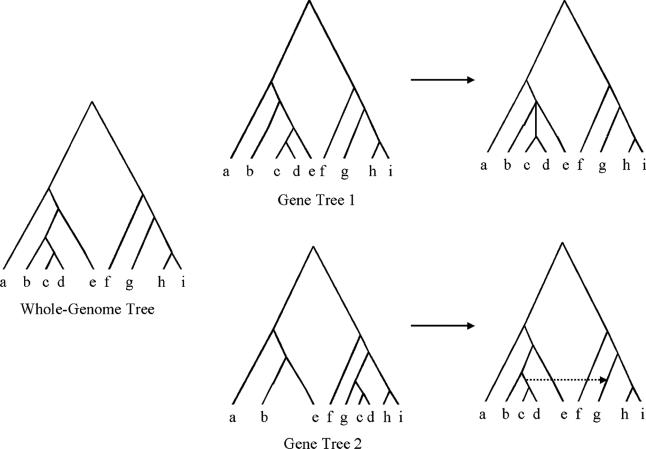
HGT Inference via Tree Comparison Raw difference between the SD and the MAST metrics for a given pair of trees tends to increase when HGT is involved in one tree. For example, the raw SD and MAST scores for Gene Tree 1 and the W-G tree are 2 and 2, respectively, while the SD and MAST scores for Gene Tree 2 and the W-G tree are 8 and 2, respectively. This difference between the SD and the MAST scores indicates possible HGT in Gene Tree 2; the (c and d) clade are transferred to the g lineage (dotted arrow). In Gene Tree 1, the (c and d) clade cannot be inferred as transfers because many other factors could have caused the local uncertainty in branching, which should be presented in polytomous form.

Taking this into account, we developed a hypothesis test for HGT, using the difference between the normalized values of the two metrics, which we denote by γ (see Materials and Methods). We computed the significance of an observed γ-value by generating a nonparametric null distribution based on randomly bootstrapped gene trees (see Materials and Methods). In our tree topology-based HGT test, we do not explicitly take branch length into account; however, the bootstrap distribution implicitly allows the incorporation of branch-specific confidence. HGT was inferred when the observed γ was significant with the *p*-value below the 5% level. The power of this procedure in detecting HGT was tested with a simulation study (detailed in Materials and Methods). These simulation studies applied to each COG showed that on average we were able to detect HGT events at 53.8%, 70.0%, and 77.3%, respectively, for one, two, and three HGT events in a COG tree using the 5% significance value. That is, if the tree contains two HGT events, we can detect the event 70.0% of the time, while guarding against false positive error at the 5% level. We examined the power of our procedure individually for each of the COG datasets; however, we did not observe a significant difference in power between those COG entries where we actually detected HGTs and those where we did not. Therefore, the procedure is not biased toward estimating HGT for one particular kind of tree over another. We also examined the effect of the number of genomes in each COG. Figure 4 shows the results, where the power increases somewhat with larger COG entries, but remains relatively stable. In particular, for those cases where we detected significant HGT events, we do not see a bias toward larger COG entries.

**Figure 4:**
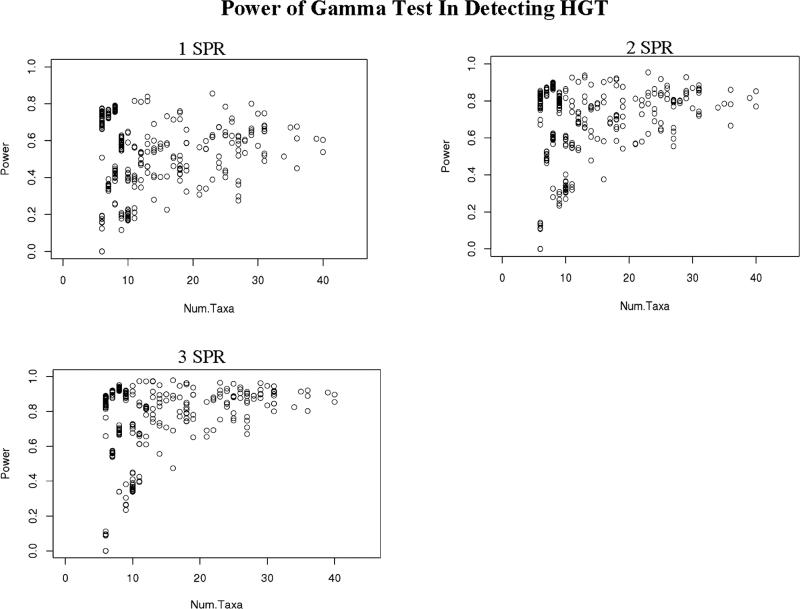
Power of the γ Test in Detecting HGT Random SPR operations were applied to each COG tree to assess the power of the γ test. The figures show the power values plotted against the taxon numbers in the COG entries for 1, 2, and 3 SPR changes.

### HGT Estimation via Comparisons between Each Gene Tree and the W-G Tree

The hypothesis test described above was applied to each of the 297 COG gene trees against the W-G tree. We expected different *p*-values for the significance level to affect the power of the test, with larger critical *p*-values tending to more liberally infer HGT events. We investigated the effect of the significance levels on the inferred number of HGT events. The number of COG entries inferred to contain HGT events does not increase dramatically as the cutoff significance value increases (Figure 5). Thus, assuming the standard 5% significance level seemed acceptable to guard against type I error; more liberal values are not expected to significantly change our conclusions about genomic rates of HGT. At the significance level of 0.05, we inferred that 33 out of 297 COG entries (i.e., 11.1%) contain putative HGT events (Table 2). Below, we will call the COG entries with statistically significant HGT events hCOGs. These hCOGs cover a wide range of functional categories as annotated in the COG database [36]. Figure 6 shows the relative frequency of hCOGs within each functional category and aggregated into broader functional categories. We used Fisher's exact test (two-sided) [46] to determine the relationship between the presence of HGT and functional categories. Only one functional category H (coenzyme metabolism) stood out as having a significantly higher (at 0.05 significance level) amount of HGT events. This is in agreement with HGT cases found in literature [4] and supports the speculation that so-called operational genes are more prone to HGT than so-called informational genes [24,47].

**Figure 5:**
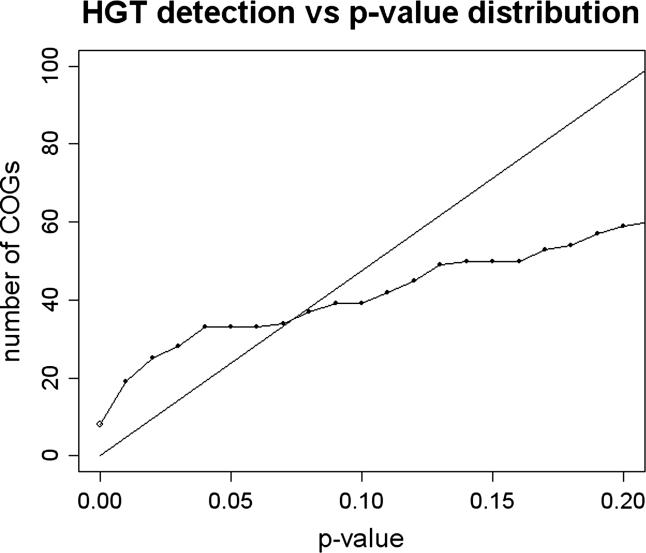
The Relationship between Detecting COG Entries with HGT and the *p*-Values Dotted curve: the number of COG entries detected to contain HGT at given *p*-value cutoffs. Straight line: the number of COG entries identified to contain HGT merely by chance, based on given *p*-value cutoffs. When the cutoff for *p*-value increases, the number of COG entries that might contain HGT increases, as one would expect. However, the small slope of this curve compared with the line of null hypothesis suggests that the frequency of HGT does not change dramatically, even in a relatively flexible *p*-value range.

**Figure 6:**
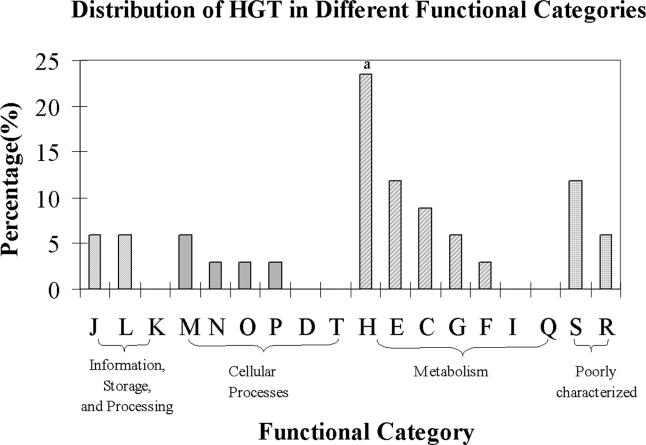
Distribution of Transferred Genes in Different Functional Categories Functional category abbreviations can be found in Table 2. The percentage of transferred genes in coenzyme metabolism (H) is significantly high, based on Fisher's exact test.

**Table 2:**
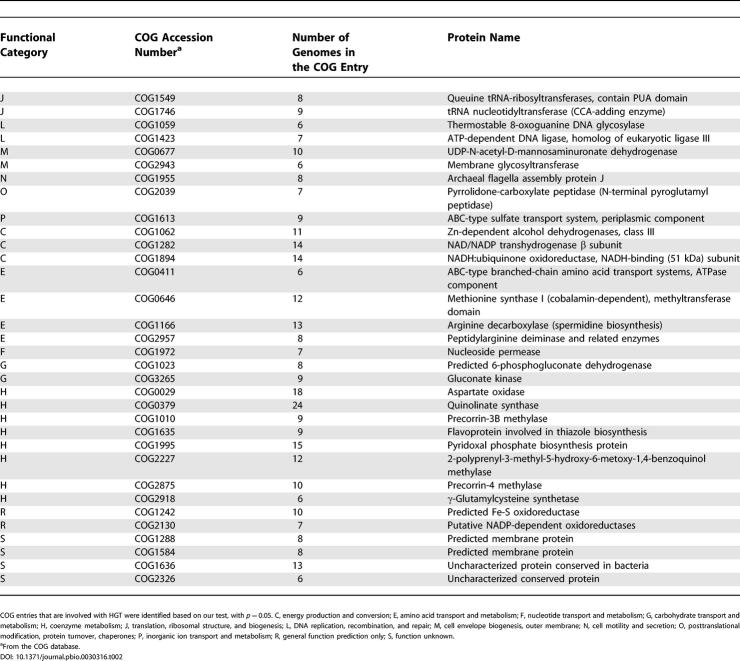
List of Transferred Genes

### HGT Estimation via Comparisons among Gene Trees

One problem with the above procedure is that the results are sensitive to the particular reference tree, i.e., the W-G tree. To overcome this problem, we next tested for possible HGTs by all pairwise comparisons of 297 COG entries. However, the COG entries do not all share the same taxa, and when the number of shared taxa is too low, we do not have sufficient power to estimate HGTs. Thus, we compared 14,004 pairs of gene trees that contained greater than or equal to six shared taxa. The same hypothesis test for HGT was applied to these pairs of gene trees. With the significance cutoff at 5% level, 1,764 out of the 14,004 pairs were significant under our test, suggesting that 12.6% of the tree pairs contain two trees significantly different from each other in terms of HGT. We then used this fraction to calculate the percentage of hCOGs. In pairwise comparisons, we have the following four cases: (1) neither tree has HGT events; (2) the first tree has HGT events; (3) the second tree has HGT events; and (4) both trees have HGT events. Suppose in our collection, we have *x* percent of COG entries with detectable HGT events. Then for a given COG, if it is a normal COG, we would expect it to test significantly different in *x* percent of the comparisons; if it is an hCOG, it should test differently for all of the comparisons. By considering such pairwise tests we can estimate the percentage of the COG entries with detectable HGT events (see Materials and Methods for more details). In our case, we estimate that 13% of COG entries may contain HGT, which is not far from the estimate (11.1%) obtained from W-G tree comparison. The pairwise test may have greater power for discrimination since most of the gene trees are fully resolved compared to our W-G tree.

### HGT Frequency in 40 Microbial Genomes

For each of the 33 hCOGs that were identified based on the comparison between each COG tree and the W-G tree, we estimated the positions of putative transfers by using an exhaustive searching procedure (see Materials and Methods for details). This allowed us to compute the genome-specific rate of HGT events among the high-quality COG entries as an estimate of the overall rate of HGT events per genome. Figure 2 shows a colored annotation of the genome-specific rate of HGT laid on top of the W-G phylogeny. Table 3 lists the HGT rate per each genome and the particular COG entries involved in the HGT. The distribution of HGT events along the W-G phylogeny shows no obvious pattern of concentrated events: genomes with high rates of HGT events seem evenly scattered across the phylogeny. As listed in Table 3, the frequency of HGT events ranges from 0% in *Chlamydia pneumoniae* and six other genomes to 6.7% in *Methanobacterium thermoautotrophicum.* The rates of HGT in *Aeropyrum pernix, Xylella fastidiosa,* and some other archaeal organisms, which are notable for their dynamic genome evolution, are relatively high in our result; while the rates for some intensely studied organisms, such as *Escherichia coli,* are not as high as previously reported [17,20]. Of the top five genomes in our list, all except the *M. thermoautotrophicum* rank highly for rates of HGT in other surveys [e.g., 17,48,49]. *M. thermoautotrophicum,* which seems to be typically at the middle of HGT rates in other surveys, stands out in our assay. One possibility is that Dufraigne et al. [48] found *M. thermoautotrophicum* to have unusually long stretches of putative HGT tracks—and perhaps offering more power by our topology-based test. The mean rate of HGT, 2.0%, among core genes per genome, is considerably lower than those reported in other studies, but the result is consistent with some phylogenetic studies focusing on smaller sets of species [50].

**Table 3:**
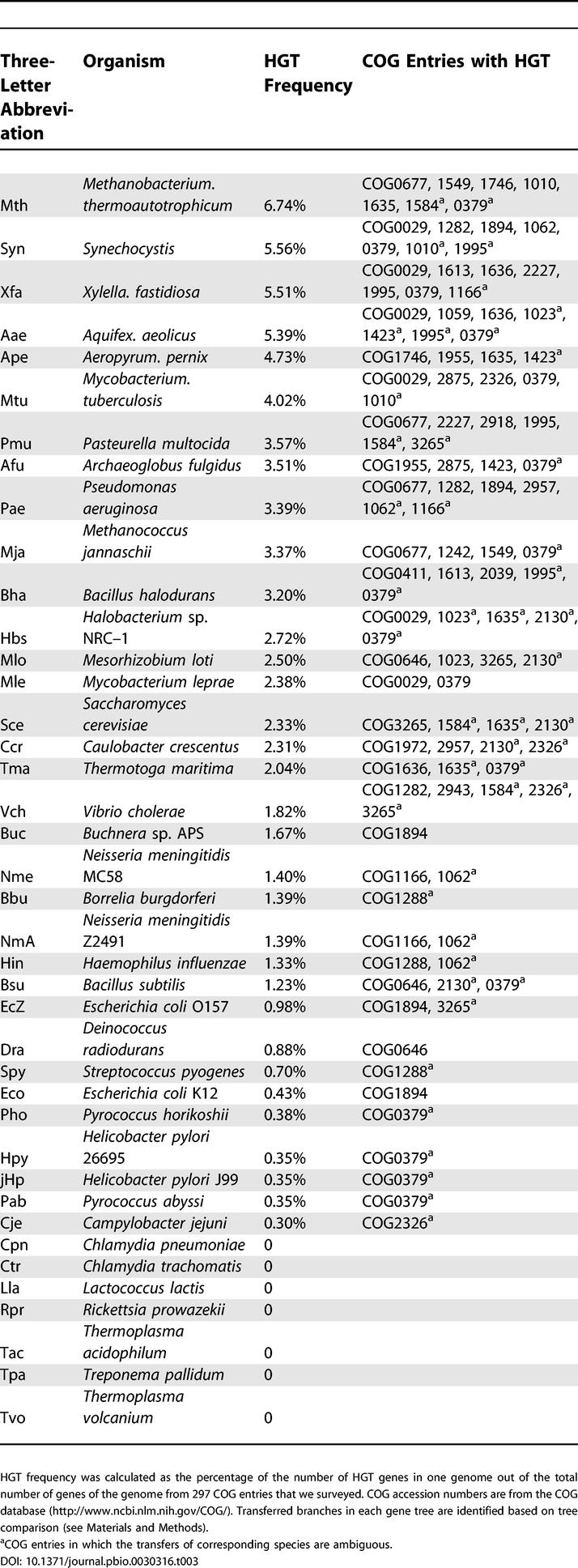
Frequency of HGT in 40 Genomes and List of Transferred Genes

## Discussion

Our main results show that HGT events can be inferred in only 33 out of the 297 COG entries studied (11.1%) in a comparison against a reference tree and 13% in pairwise comparisons among the tree pairs. The estimated rates of HGT in different genomes are between 0% and 6.7%, with an average of 2.0% among the 40 genomes studied here. There are several factors to consider in this rate estimation. First, as noted in Daubin et al. [51], one of the key questions is the rate of HGT events within those genes that can be orthologously compared to one another reliably (even if they are part of a paralogous family). The use of the COG database and our procedure for retaining only high-quality COG entries mean that our rate computation is limited to such gene sets. Thus, similar to Lerat et al. [28], where very few conflicts among gene trees of widespread single-copy orthologs in γ-Proteobacteria were found, our computed rate of HGT is only for those genes for which reliable orthologous copies can be found in multiple genomes. This might underestimate the HGT rates by ignoring sporadically distributed genes shared by only two or three genomes and those orthologous groups that cannot be reliably assembled via the mutual best-hit approach. On the other hand, for genes from large paralogous families or those only found in a few genomes, reliable assessment is impossible for either HGT or vertical transmission.

Second, we used a specific statistical test where, rather than simply asking whether two gene trees are significantly different from each other, we asked whether the trees are different and can be significantly better explained by horizontal transfer. With this in mind, we conducted a test for a specific alternative hypothesis of HGT rather than the broad rejection of simple tree congruence. A specific test of alternative hypothesis provides additional protection against false rejection of the null hypothesis. Our simulation studies suggest that our test retains reasonable power for detecting HGT events despite this additional precaution. Recently, Novichkov et al. [35] carried out a test for abnormal pairwise divergence patterns similar to our test (but with a stronger assumption of a molecular clock) and found possible HGT in approximately 17%–30% of the COG entries. The fact that we specifically test for positive evidence of HGT and allow more relaxed non-clock-like evolution may explain this discrepancy.

Third, the significance level of the hypothesis test can change the rate estimates. However, within the range of values examined, the estimated numbers of HGT events do not significantly change with increased risk of false rejection. For example, if we increase the significance value to 0.1, then we obtain 39 out of 297 COG entries (13.1%) that may contain HGT events, which is still within the lower range of values reported by others.

Fourth, our statistical test has greater power for phylogenetically distant transfer events compared to proximal transfers. This is because the tree comparison metric SD and MAST differ the most when a tree involves a branch transfer among distant taxa. For nearest-neighbor branch transfers, both methods yield the same value and thus cannot distinguish simple statistical error versus potential HGT event. Hence, if the HGT events frequently involve sister taxa, our estimate of HGT rates will be an underestimate. It is not clear whether HGTs should be more common between close lineages [52]. The mechanism and potential effect of HGT events are different from recombination and hybridization, and therefore it is difficult to assert a lineage distance effect. For example, HGT between distantly related taxa might be argued to be more likely purely due to the increased elapsed time.

Finally, we excluded the high SD and high MAST as cases where HGT events cannot be decided with high confidence. We tested the significance of both high SD and MAST scores using the bootstrap procedures described above. We found just 44 out of 297 cogs (14.8%) that have significantly high SD and MAST values but do not have significantly low γ for both the W-G tree comparison and the pairwise comparison. We are wary of treating such cases as HGT events, but regardless, these cases can be considered to add to an upper bound to HGT estimation. But we believe that such HGT events will be very difficult to detect based on gene genealogies alone. A reliable test would require more densely sampled taxa or other supporting evidence such as sequence compositional characteristics.

We have previously shown by simulation methods that even when there are large-scale HGT events (several events per gene), there remains a recognizable tree that represents the consistent treelike evolution of the majority of the genes and lineages [34]. One way to consider this is to imagine a very large tree, say 10,000 taxa, and some large number of potential “units” of HGT, say 10,000 such elements per genome. Even if each such element had, say, 1,000 actual HGTs across the 10,000 taxa, if we overlay the 10,000 trees on top of one another, all the HGTs will appear as extremely thin connections like cobwebs, and we will see a strong image of a backbone tree. More precisely, consider the relative distance between two taxa as estimated by a set of *n* genes. Assume that our estimators are perfect; we can obtain exact scaled distance estimates in such a manner that we can estimate the absolute time of separation of the two cell lineages. Let the true time of separation be *T* ^0^ and assume that an HGT event along the two lineages yields some variant time estimate, larger if the HGT event brings in a homologous copy from outside the extent of the two lineages, or smaller if the HGT event involves homologous copies from inside the two lineages. Say, for the *n* genes, *k* of them experienced horizontal transfer; then we have (*n* − *k*) values of *T* ^0^, and we need at least as many coincident draws for the HGT time estimates to set some other time estimate to be the modal value—an extremely unlikely event given the possible variant time points in a diverse tree. Thus, while an HGT event can considerably distort the treelike structure of genomic information, there still remains a distinct tree representing the modal information lineage.

Our W-G tree described here is an explicit estimation of this “modal lineage” tree. The estimation of such a modal lineage tree allows us to use explicit tree-based techniques to estimate deviations, i.e., HGT events. When we dissect the signals based on phylogenetic methods with an explicit hypothesis test for HGT, we find that HGT is not as widespread as previously believed [53,54]. Furthermore, the estimated degree of HGT is consistent no matter whether we base it on the modal lineage tree or on pairwise comparisons. The list of HGT candidates is far from being long enough to be called “rampant” for orthologous gene sets, and the overall rates are similar to those found in other studies using phylogenetic methods [28]. We are far from claiming that the reconstruction of the history of life is trivial; however, new developments in orthologous clustering, multiple sequence alignment, tree construction algorithms, and tree-rooting problems may shed more light on the impact of HGT on phylogeny and help us understand the multiple forces of prokaryotic evolution.

## Materials and Methods

### Input data preparation—Selecting high-quality COG entries

We obtained a set of putative orthologous gene clusters from the COG database (the initial version [36]; ftp://ftp://ftp.ncbi.nih.gov/pub/COG/old/). These data were processed in the following way to increase the reliability of later analysis. (We also excluded three small genomes from the original COG database, as the number of high-quality COG entries covering these genomes was too small.) (1) Best-hit confirmation: all-against-all BLAST searching was redone for all 43 genomes, and every “two-way or one-way best-hit” status for each pair of proteins was tested. Protein members that were not the top hits for any other proteins were removed from the dataset. (2) Removal of large protein families: some of the COG entries are superprotein families, which have gone through extensive gene duplication. They are not easy to use in building reliable sequence alignments and are not suitable for supertree construction. We excluded those large COG entries where the number of sequences exceeds the number of genomes by more than 2.5-fold. (3) Estimation of COG quality by checking BLAST sequence alignments: the quality of each COG was assessed based on the pairwise BLAST *e*-values and lengths of the significant aligned regions. We obtained 511 COG entries from which all the *e*-values of pairwise BLAST scores were lower than 10^–10^, and whose proportion of high-scoring aligned regions to the whole protein sequences was greater than 50%. (4) Building distance matrices: we first generated multiple sequence alignments using CLUSTALW [55], and then used PHYLIP [56] to calculate distance matrices based on the alignments. PHYLIP's command tool “prodist” with default setting of Dayhoff PAM matrix was used to make our calculations. Gap regions in the alignments were dropped because they might not have been aligned properly. (5) Exclusion of paralogs from each COG: in order to have only one representative gene from one genome in each COG, we removed putative paralogous genes. Based on the distance matrix, if the distance between paralogs within one genome was less than the distance to homologs in other genomes (so-called in-paralogs), we randomly chose one of them. If the two paralogs were not closer to each other than to other homologs from other genomes, we filtered out the two paralogs of the same genome from our analysis because they conflicted with each other. After this step, we retained 297 COG entries with at most one sequence per genome. At the same time, these COG entries contain at least six taxa.

### Building gene trees and the W-G tree

For each of the 297 COG entries we constructed a gene tree by computing a neighbor-joining tree (PAUP* [57]), using the distance matrix computed as described above. For each gene tree, 1,000 bootstrap replicates were computed by bootstrap sampling from the original sequences and computing a replicate distance matrix. We computed a consensus tree for the bootstrap replicates according to the majority rule; this was used as the gene tree estimate.

We then applied the median tree algorithm [34] to 230 out of the 297 COG entries to build the W-G tree estimate. First, to describe in brief, given a set of distance matrices, the algorithm computes the median of normalized distances as a robust estimate of the true evolutionary distance. It has been shown to be particularly useful for estimating the genome tree when individual genes undergo HGT events. The detailed procedure follows: (1) Data selection: although there were 297 high-quality COG entries, those that covered only a small number of genomes could render the normalization process unstable. Therefore, we used a subset of 230 high-quality COG entries that covered at least seven genomes for the W-G tree estimate. (2) Normalization of distance matrices of COG entries: the median tree algorithm requires us to normalize the distance matrices of COG entries so that we can minimize the difference in evolutionary rates of all the genes in different COG entries. We carried out the normalization to get a scaling factor for each COG in three steps: (i) we selected a single COG that covers all 40 genomes as the reference COG (see Accession Numbers section); (ii) for each COG, we calculated the ratio of pairwise distance for each two genomes to the corresponding pairwise distance in the reference COG; (iii) we used the median of the ratios of the pairwise distances for each COG as the scaling factor for that COG. (3) Building the median tree: for each pair of genomes, the genomic distance was defined as the median of the normalized distances between this pair of genomes for all the 230 normalized COG entries. The median distance of each pair of genomes was retrieved from an average of 45 COG entries, with a minimum of six and a maximum of 147 COG entries (standard deviation = 25). Each COG contributed to an average of 19.7% of the entries in the final median distance matrix, with a minimum of 3.6% and a maximum of 100% (standard deviation = 21.0%). The median distance matrix calculated in this manner was used in conjunction with PAUP* [57] to construct a neighbor-joining tree. One thousand bootstrap replicates of the W-G tree were obtained by bootstrap resampling of the 230 COG entries (the reference COG was guaranteed to be in each resampling), recomputing the median distance matrix, and applying the neighbor-joining method. The majority-rule consensus tree of the bootstrap replicates was computed to estimate the W-G tree (see Figure 2).

### HGT inference and power testing

HGT events were tested by computing a statistic γ based on tree topological comparisons. For a pair of trees *T* and *T*′, with *m* and *n* splits (i.e., branches), respectively, and with *x* number of taxa, our statistic γ is defined as:



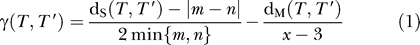



where *d_S_* is the SD metric [45], and *d_M_* is the MAST metric [44], both of which can be calculated using PAUP* [57]. The terms on the right-hand side are normalized values of the SD and the MAST metrics. The normalization takes into account the effect of the size of the trees on SD and MAST metric. The null distribution described next is also based on the normalized statistics, thus controlling for taxon sampling effects of tree topologies.

The null distribution of γ was obtained by a randomization procedure. For each COG, 2,000 bootstrap trees were generated from the original sequence alignment. We divided them into 1,000 pairs of trees, for each of which the statistic γ was calculated. The distribution of 1,000 γ-values computed in this manner represents the null distribution in which the tree differences are not due to HGT. For each COG, γ was calculated for the gene tree against the W-G tree in the W-G approach or against another gene tree in the pairwise comparison approach, with both trees pruned to the same set of taxa. If the γ-value for a COG was higher than 95% of γ-values for bootstrapped trees, we accepted the alternative hypothesis that HGT is present.

We used random subtree pruning–regrafting (SPR) operations [58] on each COG tree to assess the power of the test, since a gene tree with an HGT is the result of applying a corresponding SPR to the W-G tree. We applied one to three random SPR operations on the COG tree to obtain a changed tree. We repeated this experiment 10,000 times for each combination of COG entries and the number of SPR operations; in this manner we collected the distribution of the γ statistic under the alternative hypothesis of HGTs. This allowed us to obtain the power of our test based on a 0.05 significance level. The average power values for all COG entries are plotted against the number of taxa in the COG entries (see Figure 4). While the power clearly increases with the size of COG entries, the size of the hCOGs does not show a biased distribution, and thus differential power with respect to the numbers of taxa does not seem to be a factor in our results.

### Testing the relationship between HGT and functional categories**

For each functional category, a 2 × 2 contingency table was built with four elements: (1) number of COG entries with HGT in this category; (2) number of COG entries without HGT in this category; (3) number of COG entries with HGT that are not in this category; (4) number of COG entries without HGT that are not in this category. Two-sided Fisher's exact test was applied to test the association between the HGT and functional category.

### Estimation of fraction of COG entries with HGT based on pairwise COG tree comparison

The 297 COG entries may contain overlapped taxa. We compared 14,004 pairs of COG trees that had at least six shared taxa via our γ test. If a given COG contained HGT, we would expect this COG to test positive against all other comparable COG entries. If a given COG is normal, it will only test positive against some unknown fraction *P* of the hCOGs. Therefore, for each COG we applied our γ test against all other comparable COG entries with a Bonferroni correction for multiple tests. If a COG tested positive against greater than some predetermined *P* percent of hCOGs, that COG was assigned to the hCOG category. After applying this procedure to the entire set of COG entries, the fraction hCOGs was computed as the value *Q.* If our procedure is consistent, we should obtain *P* = *Q.* Therefore, we iterated through all values of *P* and repeated our process until *P* = *Q,* resulting in an estimated 13% of COG entries ending up in the hCOG category.

### Identification of transferred branches in gene trees

The comparison between a gene tree and the W-G tree described above infers presence and absence of HGT events for a given COG. For each COG that tested positive for HGT events, we identified the particular branches of transfer by exhaustive enumeration of possible subtree matches. Since the MAST score gives the number of taxa needed to make the two trees identical, we exhaustively searched for all combinations of branch prunings to find the “troublesome” branches. When there is only one way of pruning branches to make the two trees congruent to each other, those pruned branches are identified as HGT events. However, on a limited number of occasions, there was more than one way of pruning the branches. We treated those branches as equally probable transfers and assigned them a probability weight based on the number of possible prunings. For each genome, the total number of putative HGT events was summed, and the rate of HGT was calculated based on the number of hCOG entries that contained genes from that genome.

## Supporting Information

### Accession Number

The COG database (http://www.ncbi.nlm.nih.gov/COG/) accession number for the reference COG is COG0541.

## Abbreviations

COG: Clusters of Orthologous Groups
hCOG: Clusters of Orthologous Groups database entry containing horizontal gene transfer
HGT: horizontal gene transfer
MAST: maximum agreement subtree
SD: symmetric difference
SPR: subtree pruning–regrafting
W-G tree: whole-genome tree
